# Characterization of the Modes of Binding between Human Sweet Taste Receptor and Low-Molecular-Weight Sweet Compounds

**DOI:** 10.1371/journal.pone.0035380

**Published:** 2012-04-20

**Authors:** Katsuyoshi Masuda, Ayako Koizumi, Ken-ichiro Nakajima, Takaharu Tanaka, Keiko Abe, Takumi Misaka, Masaji Ishiguro

**Affiliations:** 1 Suntory Institute for Bioorganic Research, Mishima-gun, Osaka, Japan; 2 Department of Applied Biological Chemistry, Graduate School of Agricultural and Life Sciences, The University of Tokyo, Bunkyo-ku, Tokyo, Japan; 3 Food Safety and Reliability Project, Kanagawa Academy of Science and Technology, Kawasaki, Kanagawa, Japan; 4 Niigata University of Pharmacy and Applied Life Sciences, Akiha-ku, Niigata, Japan; German Institute for Human Nutrition, Germany

## Abstract

One of the most distinctive features of human sweet taste perception is its broad tuning to chemically diverse compounds ranging from low-molecular-weight sweeteners to sweet-tasting proteins. Many reports suggest that the human sweet taste receptor (hT1R2–hT1R3), a heteromeric complex composed of T1R2 and T1R3 subunits belonging to the class C G protein–coupled receptor family, has multiple binding sites for these sweeteners. However, it remains unclear how the same receptor recognizes such diverse structures. Here we aim to characterize the modes of binding between hT1R2–hT1R3 and low-molecular-weight sweet compounds by functional analysis of a series of site-directed mutants and by molecular modeling–based docking simulation at the binding pocket formed on the large extracellular amino-terminal domain (ATD) of hT1R2. We successfully determined the amino acid residues responsible for binding to sweeteners in the cleft of hT1R2 ATD. Our results suggest that individual ligands have sets of specific residues for binding in correspondence with the chemical structures and other residues responsible for interacting with multiple ligands.

## Introduction

**Figure 1:**
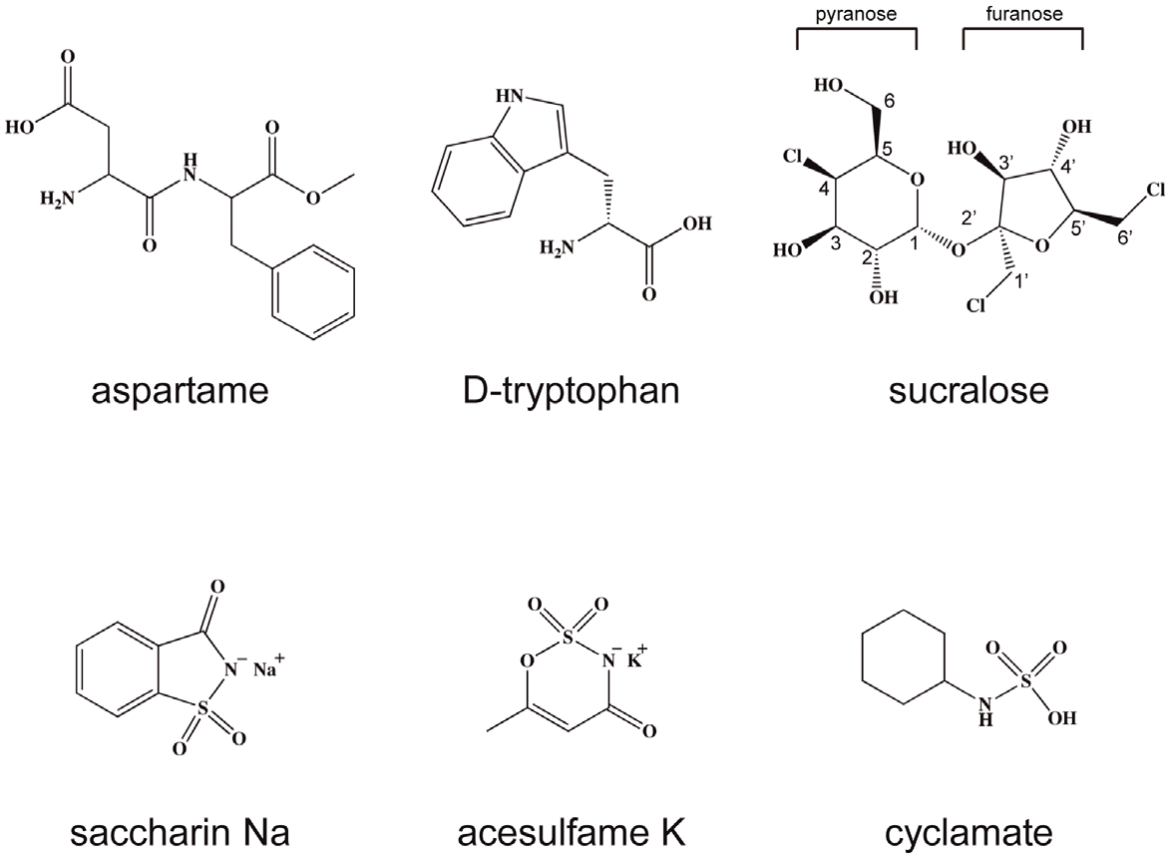
Chemical structures of the small molecular sweeteners used in this study.

The human sweet taste receptor (hT1R2–hT1R3) is a heteromeric complex composed of two subunits, T1R2 and T1R3, which are class C G protein–coupled receptors (GPCRs) [1], [2], [3]. Each subunit has a large amino-terminal domain (ATD) linked by an extracellular cysteine-rich domain (CRD) to a seven-transmembrane helical domain (TMD) [4]. hT1R2–hT1R3 responds to a wide variety of chemical substances including naturally occurring sugars (glucose, sucrose, fructose and sugar alcohols), D-amino acids (D-tryptophan and D-phenylalanine) and glycosides (stevioside and glycyrrhizin), as well as artificial chemical compounds such as sucralose, aspartame, neotame, saccharin Na, acesulfame K (AceK), and cyclamate (Fig. 1) [5]. Moreover, naturally occurring sweet proteins, such as brazzein, thaumatin, and monellin, and naturally occurring taste-modifying proteins, such as neoculin and miraculin, also bind to hT1R2–hT1R3 [6], [7], [8], [9], [10], [11]. hT1R2–hT1R3 has multiple ligand-binding sites for these various sweeteners. For example, the ATD of hT1R2 is responsible for binding to aspartame and sugar derivatives [9]. Neoculin binds the ATD of hT1R3 [12]. In contrast, cyclamate and neohesperidin dihydrochalcone (NHDC) bind the TMD of hT1R3 as agonists [13], whereas this region also serves as the allosteric binding site for saccharin and lactisole as antagonists [14].

The structural features of the ATD of the homodimeric metabotropic glutamate type 1 receptor (mGluR1) have been identified by X-ray crystal structure analysis, and this was the first example to reveal the structure of a class C GPCR [15]. The ATD of mGluR1 comprises two lobes (LB1 and LB2) that form the glutamate-binding domain lying between LB1 and LB2. The structure of ATD exists in an equilibrium of two different conformations, and the structural change strongly depends on glutamate binding. In the ligand-free state, both LB1 and LB2 tend to show open conformations (open-open), whereas an agonist induces a closed conformation for LB1 and LB2 of one ATD, while the other remains in an open conformation. This closed-open structure is thought to contribute to the active state of mGluR1 [15].

Because hT1R2 and hT1R3 share sequence homology (24% and 23%) with mGluR1 (Fig. S1), they also share some common structural features with mGluR1 [16]. hT1R2–hT1R3 can form a heterodimer, with the open-open form representing an inactive structure and the closed-open form representing an active structure. When low-molecular-weight sweeteners are applied, hT1R2 probably exhibits a closed conformation because the ATD of hT1R2 receives aspartame and sugar derivatives [17], [18]. Not only these small sweeteners but also cyclic sulfamate derivatives such as saccharin sodium and AceK probably bind at the cleft formed by LB1 and LB2 of hT1R2 ATD; they differ from each other in their hydrophobicity, electric charge, molecular size and other parameters (Fig. 1). Naturally occurring hydrophilic sugars are generally different in chemical structure from rather hydrophobic artificial amino acid derivatives and cyclic sulfamate derivatives. Moreover, amino acid derivatives and cyclic sulfamate derivatives have charged groups, whereas sugar derivatives are neutral.

Several ligand-binding sites were proposed by a molecular modeling–based docking simulation for the sweet taste receptor [6], [8], [11], [16], [19]. Thus, the wedge site of an open form of the ATD of the T1R3 was proposed for sweet proteins [6], [8], [16], whereas the involvement of the CRD of the T1R3 was proposed for brazzein, a sweet protein [11]. On the other hand, the cavity of the closed form formed by LB1 and LB2 of either T1R2 or T1R3 [11], [16] is suggested for small sweeteners as glutamate bound in the glutamate receptor [15]. In this study, we found that the various structures of low-molecular-weight sweeteners were recognized by the sweet taste receptor hT1R2–hT1R3 through the different residues at the ligand-binding site of the ATD of T1R2. Modes of binding between hT1R2–hT1R3 and low-molecular-weight sweet chemical substances were characterized both by response profiles of cells expressing the mutated hT1R2–hT1R3 to sweeteners and by a molecular modeling–based docking simulation at the binding cleft formed by LB1 and LB2 of hT1R2. The candidate amino acid residues at the binding cleft of hT1R2 were targeted to produce mutated hT1R2, which was then heterologously expressed in cultured cells together with hT1R3 and its coupling Gα protein. Using the functional analysis of cell-based assays, we successfully determined the residues responsible for binding to each sweetener in the ligand-binding cleft of hT1R2 ATD and found that individual molecules use characteristic residues for binding. A mechanism of receptor activation is also discussed according to a molecular model of the receptor–ligand complex.

## Materials and Methods

### Site-directed mutagenesis of hT1R2 cDNA

cDNA fragments with point mutations in hT1R2 were synthesized by the overlap PCR method using mutated primer pairs. The following 15 residues in hT1R2 were mutated individually to Ala: S40, K65, Y103, D142, S144, S165, Y215, P277, D278, Y282, E302, S303, D307, E382, and R383. In the cases of Y103, D142, Y215, P277, and R383, each residue was also replaced with residues other than Ala (Y103F, D142R, Y215F, P277G, P277Q, P277S, R383D, R383Q, R383L, and R383H).

### Calcium imaging analysis of the heterologously transfected cultured cells

cDNA fragments were subcloned into the pEAK10 vector (Edge Biosystems, Gaithersburg, MD, USA). Each hT1R2 mutant was transiently cotransfected together with hT1R3 and G16-gust44 [20] into HEK293T cells (kindly provided by Dr. Hiroaki Matsunami, Duke University), and calcium imaging analysis was carried out as described previously [12]. Briefly, transfected cells were seeded into 96-well Lumox multiwell black-wall plates (SARSTEDT AG & Co., Nümbrecht, Germany). After 40–46 hours, the cells were loaded with 5 µM of fura-2/AM (Invitrogen, Carlsbad, CA, USA) in assay buffer for 30 min at 37°C, and then washed with assay buffer, prior to incubation in 100 µl of assay buffer for more than 10 min at room temperature. The cells were stimulated with sweet tastants by adding 100 µl of 2× ligands. The intensities of fura-2 fluorescence emissions resulting from excitation at 340 and 380 nm were measured at 510 nm using a CCD camera. The images were recorded at 4 sec intervals and analyzed using MetaFluor software (Molecular Devices, Sunnyvale, CA, USA).

### Construction of stable cell lines expressing the mutated human sweet taste receptor

The entire coding regions of hT1R2, hT1R3, and G16-gust44 were subcloned into the pcDNA5/FRT vector (Invitrogen) according to the procedure described previously [21]. To generate the expression plasmid for the mutated receptor, the hT1R2 cDNA fragment with the point mutation was used instead of using the wild-type (WT) hT1R2 cDNA.

Stable cell lines expressing mutant hT1R2 together with hT1R3 and G16-gust44 were generated to prepare the following hT1R2 mutants: Y103A, Y103F, D142A, S144A, S165A, P277A, P277G, P277S, P277Q, D278A, E302A, D307A, E382A, and R383H. The stable cell lines were generated using Flp-In 293 cells (Invitrogen) and the plasmid we constructed according to the manufacturer's protocol for the Flp-In pcDNA5/FRT Complete System (Invitrogen) as described in our previous publication [21]. Hygromycin-resistant cells were collected, cultured, and used to measure the cellular responses to sweet tastants. The cells for these measurements were cultured in low-glucose (1.0 g/l) Dulbecco's modified Eagle's medium with 10% fetal bovine serum.

### Measurement of cellular responses by the cell-based assay

Trypsinized cells were seeded at a density of 80,000 cells per well into 96-well black-wall CellBIND surface plates (Corning, Corning, NY, USA) and 24 hours later were washed with assay buffer prior to loading with a calcium indicator dye from the FLIPR Calcium 4 Assay Kit (Molecular Devices) diluted with assay buffer. The cells were incubated for 60 min at 37°C, and measurements were made using FlexStation 3 (Molecular Devices) at 37°C. Fluorescence changes by excitation at 485 nm, emission at 525 nm, and cutoff at 515 nm were monitored at 2 s intervals, an aliquot of 100 µl of assay buffer supplemented with 2× ligands was added at 20 s, and scanning was continued for an additional 100 s. The response of each well was represented as ΔRFU (delta relative fluorescence unit) and defined as maximum fluorescence value minus minimum fluorescence value. To calculate EC_50_ values, plots of amplitude versus concentration were prepared in Clampfit Version 9.2 (Molecular Devices). Nonlinear regression of the plots produced the function:
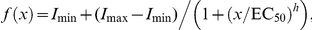
where *x* is the ligand concentration and *h* is the Hill coefficient used to calculate the EC_50_ values for ligand–receptor interactions. When the EC_50_ value of the mutated receptor-expressing cells was changed more than 5 fold compared with wild type receptor, the corresponding mutation was judged to be largely affected.

### Structure modeling of receptor and receptor–ligand complexes

The crystal structures of the ATD of mGluR1 solved in both inactive (glutamate-unbound) and active (glutamate-bound) forms (PDB: 1EWT and 1EWK, respectively) were used to construct the ATDs of hT1R2 and hT1R3. The structural model of the ATDs of the hT1R2 and hT1R3 heterodimer was constructed with homology modeling according to their sequence homology with mGluR1. For the active form of the heterodimer model, the closed form of mGluR1 was used for hT1R2 and the open form for hT1R3. Conversely, the open form of the crystal structure of mGluR1 was used to construct the inactive form of T1R2 and T1R3. Each heterodimeric structure was then energy-minimized with molecular mechanics using Discover 3 (Accelrys Inc., CA, USA), and the main chain was tethered at the conserved position.

Sweet small ligands were docked into the ligand-binding cleft of the hT1R2 model where glutamate is bound in the mGluR1; this was pursuant to the plausible interactions between the charged or hydrophilic groups of the ligands and the receptor that were deduced from the mutational experiments. Conformations of the ligands were then generated and energy-minimized with molecular mechanics using Discover 3. The minimized complex structures were then structure-optimized with molecular dynamics using Discover 3, and the residues were tethered beyond 12 Å from the ligands.

## Results

### Mutagenesis studies for screening the residues responsible for sweetener recognition

To define the binding modes of sweeteners at the cleft formed by LB1 and LB2 of hT1R2 ATD, we carried out a series of mutagenesis studies on hT1R2 ATD. First, a molecular model of hT1R2 ATD based on the ligand-binding structure of the closed form of mGluR1 was constructed. Based on the residues resided in the glutamate-binding cleft in the structure of mGluR1 ATD, 15 residues of hT1R2 were arbitrarily selected to introduce the point mutation (Fig. S1, Table S1), and 25 single hT1R2 mutants for the 15 residues were then constructed. The selected residues were almost hydrophilic, and were expected to form ionic or hydrogen bonds with the ligands. The responses to sweeteners were examined by a calcium imaging assay using HEK293T cells transiently expressing the T1R2 mutant and T1R3. Ten out of the 15 residues (Y103, D142, S144, S165, P277, D278, E302, D307, E382, and R383) were selected from the results of the 25 mutants because receptors mutated at these 10 residues retained the responsiveness and exhibited largely changed activities toward the sweeteners tested (Table S1).

As for the 10 residues, stable cell lines expressing the hT1R2 mutant and hT1R3 were constructed, and the cell-based assay was performed to determine the dose–response relationship with the half-maximal effective concentration (EC_50_) value for each sweetener. To validate the activity of each mutated receptor, we used an artificial sweetener cyclamate, which was recognized by the TMD of hT1R3, as positive controls [22]. Because all the hT1R2 mutant cell lines clearly responded to cyclamate, showing similar EC_50_ values to those expressing the WT receptor (Table 1), the mutated receptors were determined to be functionally expressed. The response profiles of the mutated receptors to the sweeteners are summarized in Table 1.

**Table 1:** Summary of point mutations in hT1R2–hT1R3.

	aspartame	d-tryptophan	saccharin Na	acesulfame K	sucralose	cyclamate
mutants	EC_50_ (mM)	EC_50_ (mM)	EC_50_ (mM)	EC_50_ (mM)	EC_50_ (mM)	EC_50_ (mM)
WT	0.75±0.11	2.09±0.43	0.19±0.07	0.54±0.16	0.08±0.02	2.56±0.46
E302A	No response	No response	*0.10±0.03*	*0.35±0.14*	**0.46±0.11**	*3.53±0.91*
S144A	No response	*9.85±3.61*	*0.36±0.06*	*0.94±0.13*	*0.27±0.03*	*4.16±0.42*
D142A	No response	**12.30±4.34**	No response	No response	**6.03±4.73**	*5.83±0.99*
Y103A	No response	**15.41±7.35**	*0.64±0.09*	*1.67±0.34*	No response	*11.63±2.02*
D278A	**6.12±3.50**	**29.42±14.42**	*0.29±0.10*	*0.95±0.28*	No response	*6.53±2.40*
D307A	*3.74±0.81*	**21.27±11.56**	**1.06±0.78**	*2.16±0.31*	**1.77±0.19**	*4.14±0.25*
S165A	*0.51±0.07*	**11.31±5.19**	*0.28±0.06*	*0.60±0.13*	*0.20±0.03*	*6.19±1.87*
P277A	*1.83±0.19*	**13.21±5.50**	*0.89±0.38*	*1.61±0.32*	**2.35±0.34**	*5.12±0.70*
R383H	*1.44±0.46*	*7.39±1.80*	No response	No response	*0.30±0.03*	*6.24±2.32*
E382A	*1.76±0.60*	*4.46±2.09*	No response	No response	*0.22±0.07*	*4.45±1.27*
Y103F	No response	**20.56±8.39**	*0.45±0.26*	*0.66±0.32*	*0.34±0.03*	*4.97±0.74*
P277G	*1.12±0.29*	*8.29±1.89*	*0.62±0.29*	*1.28±0.58*	**3.22±1.87**	*5.06±1.41*
P277Q	No response	**24.29±5.67**	*0.58±0.17*	*2.02±0.70*	**6.12±3.61**	*4.75±0.70*
P277S	*1.38±0.22*	*5.82±0.94*	*0.65±0.23*	*1.55±0.39*	**1.08±0.15**	*4.04±0.68*

Effects of point mutations in hT1R2–hT1R3 on the EC_50_ values of low-molecular-weight sweeteners obtained from a cell-based assay. Each column indicates the mean ± S.E.M. from 3–5 independent experiments. Italic and bold values represent {(EC_50_ mutant/EC_50_ WT)<5.0} and {5.0<(EC_50_ mutant/EC_50_ WT)}, respectively.

### Residues responsible for aspartame and D-tryptophan reception in hT1R2 ATD

**Figure 2:**
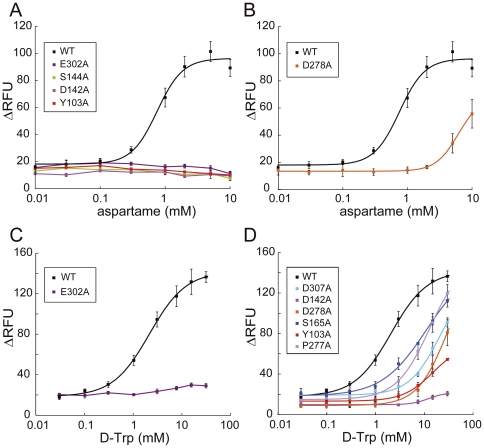
Dose-dependent responses of hT1R2/hT1R3-expressing cells to amino acid derivatives. Responses of the stable cell lines to aspartame (A, B) and d-Trp (C, D) were determined by the cell based assay. The mutations introduced into hT1R2 subunit are shown. Each point indicates the mean ± S.E.M. from at least 3 independent experiments.

The response to aspartame was completely lost in the cell lines expressing E302A, S144A, D142A and Y103A (Fig. 2A), and EC_50_ values largely increased in those expressing D278A, with a decrease in potency (EC_50_ value 8.14-fold increase versus WT, Fig. 2B). These results suggest that the residues E302, S144, D142, Y103, and D278 are crucial for aspartame reception, among which E302 and S144 have also been previously reported as important residues for aspartame recognition [17].

In contrast, only the application of d-tryptophan (d-Trp) to E302A-expressing cells elicited no response (Fig. 2C), and large increases in EC_50_ values were observed for D307A, D142A, D278A, S165A, Y103A, and P277A mutants (>5-fold increase versus WT) (Fig. 2D and Table 1). Although aspartame elicited no response in D142A and Y103A mutants (Fig. 2A and Table 1), d-Trp considerably reduced the response potency to these mutants within an 8-fold EC_50_ increase (Fig. 2D and Table 1). In the cases of S165A and P277A mutants, EC_50_ of d-Trp increases 5.40- and 6.31-fold, respectively (Fig. 2D), while those of aspartame were only changed (Table 1). Although the carboxylate of aspartame and d-Trp is located near S165 and R383 in their complex models, the carboxylate of d-Trp would interact with S165, but that of aspartame would be located at slightly different position not to directly interact with S165. A similar case is also the interactions of P277 with d-Trp and aspartame, in which the indole moiety is locate closer to P277 than the phenylalanine moiety is. The roles of S165 and P277 in receptor activation are thus ligand depended.

### Residues responsible for saccharin Na and acesulfame K reception in hT1R2 ATD

**Figure 3:**
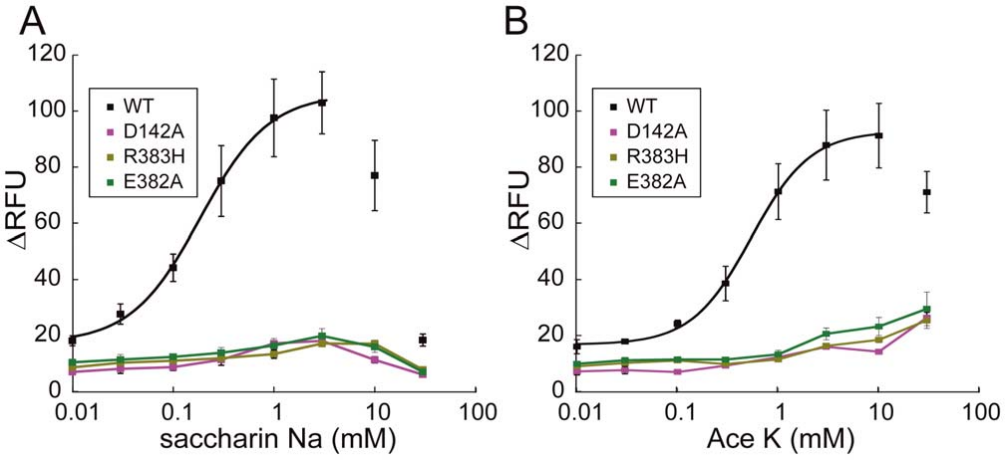
Dose-dependent responses of hT1R2/hT1R3-expressing cells to sulfamates. Responses of the stable cell lines expressing to saccharin Na (A) and AceK (B) were determined by the cell based assay, and the results of the cells expressing WT and hT1R2 mutants (D142A, R383H, and E382A) receptor are shown. Each point indicates the mean ± S.E.M. from at least 3 independent experiments.

Saccharin Na and AceK activated WT hT1R2–hT1R3 in a dose-dependent manner at lower concentrations, but the response was suppressed at higher concentrations (>3 mM and >10 mM, respectively, Figs. 3A and 3B), which has been observed and investigated in detail by Galindo-Cuspinera et al. [14]. Therefore, EC_50_ values for saccharin Na and AceK were estimated at the lower concentrations.

The cellular responses to saccharin Na and AceK were lost in R383H, D142A and E382A (Figs. 3A and 3B). These results indicate that R383, D142, and E382 are crucial residues for activation by saccharin Na and AceK. The mutations E302, S144 and D278 scarcely affected the EC_50_ values for saccharin Na and AceK, unlike aspartame and d-Trp (Fig. 2 and Table 1). Moreover, the other mutations tested in this study were not sensitive to saccharin Na and AceK (Table 1), suggesting that the binding region for saccharin Na and AceK is limited to a region around R383 (see Discussion).

### Residues responsible for sucralose reception in hT1R2 ATD

**Figure 4:**
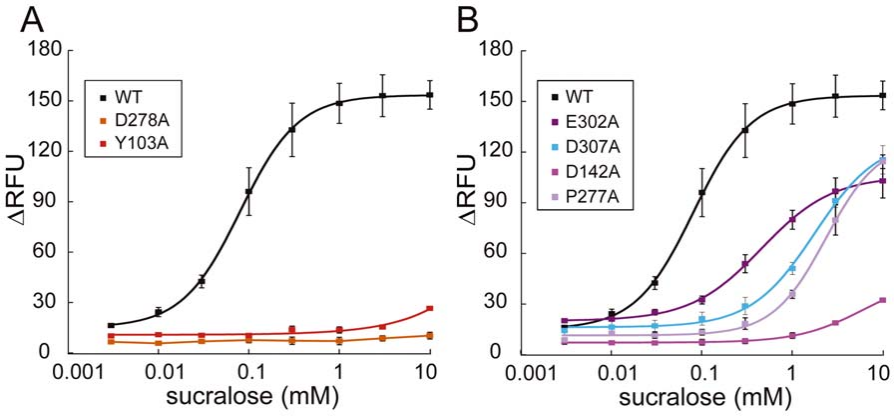
Dose-dependent responses of hT1R2/hT1R3-expressing cells to sucralose. The results of the cells expressing WT and hT1R2 mutants (D278A and Y103A) receptor are shown in A, and those of E302A, D307, D142A, and P277A mutants are shown in B. Each point indicates the mean ± S.E.M. from at least 3 independent experiments.

The response to sucralose was almost completely lost in D278A and Y103A (Fig. 4A). E302A, D307A, D142A, and P277A largely increased the EC_50_ values of sucralose and decreased the potency (Fig. 4B). Most of the crucial residues for sucralose reception (E302, D142, Y103, D278, and D307) appeared to overlap with those for d-Trp and aspartame reception (Table 1). However, unlike aspartame, the EC_50_ value of sucralose for S144A did not change dramatically (0.27 mM), and P277A elicited a remarkable increase of the EC_50_ value. These results indicate that sucralose partially shares the binding region with aspartame, but also interacts with sucralose-specific residue such as P277.

### Roles of Y103 and P277 at the entry of the lobes

**Figure 5:**
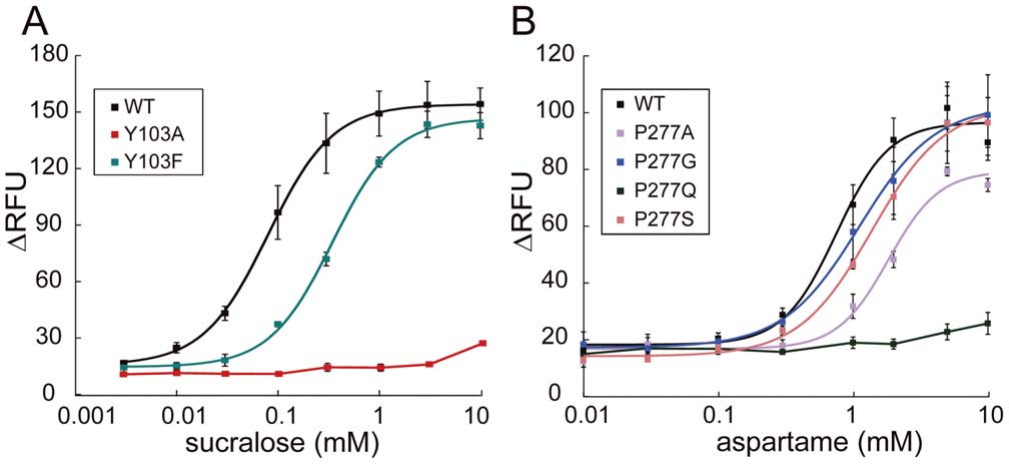
Roles of Y103 and P277 for the reception of the sweeteners. Dose-dependent responses of cells expressing Y103 mutants (Y103A and Y103F) to sucralose and P277 mutants (P277A, P277G, P277Q, and P277S) to aspartame are shown in A and B, respectively. Each point indicates the mean ± S.E.M. from at least 3 independent experiments.

Six out of the 10 critical residues (D142, D278, E302, D307, E382, and R383) are acidic or basic residues that probably bind to ligands via electrostatic interactions (Table 1). Furthermore, S144 and S165 were important for the reception of the amino acid derivatives aspartame and d-Trp, respectively (Figs. 2A and 2D). We next evaluated the role of the hydrophobic residues, Y103 and P277, located across the cleft of LB1 and LB2, respectively (See Discussion). To further examine the effect of Y103 on receptor activity, the responses of stable cell lines expressing additional mutants (in which Y103 was replaced with Phe in addition to Ala) were evaluated. When sucralose was applied to Y103 mutants, the response was almost completely lost in Y103A but was only slightly reduced in Y103F (Fig. 5A). These results indicate that the aromatic ring of Y103 is specifically essential to sucralose binding.

To evaluate the role of P277, the additional mutants P277G, P277Q and P277S were constructed. The P277Q mutant showed severely reduced responses to aspartame (Fig. 5B) and d-Trp (Table 1), while P277G and P277S did not (Fig. 5B). In contrast, these three mutants responded almost equally to saccharin Na and AceK (Table 1). These results suggest that P277 plays an important role in allowing the sweet taste receptor to discriminate amino acid derivatives (aspartame and d-Trp) from the other sweeteners.

## Discussion

### Critical residues for small molecular sweetener recognition in hT1R2 ATD

**Figure 6:**
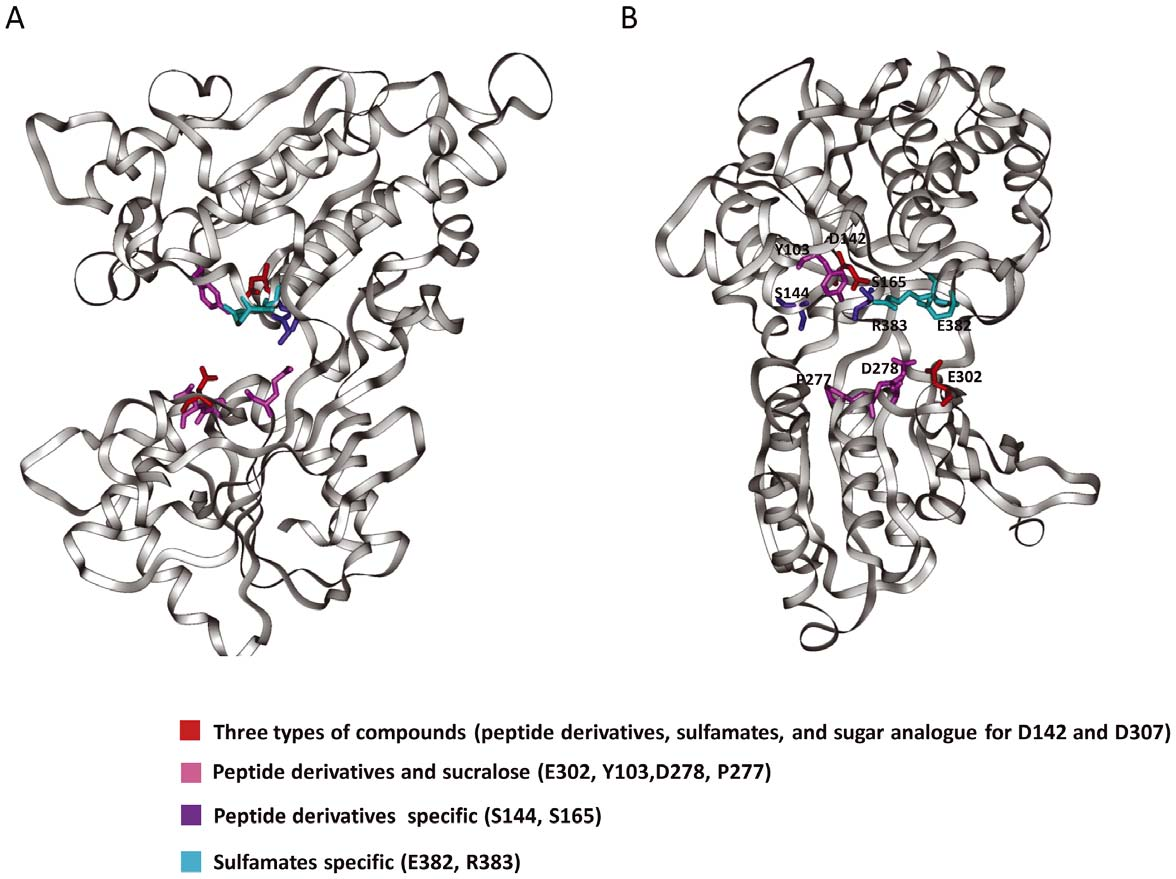
Model of the open form of hT1R2 ATD. (A) The 10 critical residues are mapped on the model as sticks. They were divided into four colors based on the results from the single point mutation analysis of hT1R2–hT1R3 using three chemically different types of ligands: amino acid derivatives (aspartame and d-Trp), sulfamates (saccharin Na and AceK), and a sugar analog (sucralose) (see also Table 1). Red: the three types of chemicals; pink: peptide derivatives and sucralose; purple: peptide derivative–specific; cyan: sulfamate-specific. (B) The model oriented 90° from (A).

**Figure 7:**
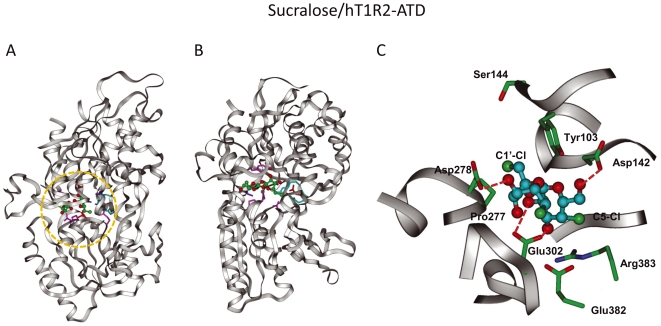
Complex model of the sucralose-bound hT1R2 ATD. (A) Complex model of sucralose in the closed form of hT1R2 ATD. Chlorine atoms are colored light green. (B) The model oriented 90° from (A). (C) Sucralose-binding pocket in detail (orange circle in (A)).

**Figure 8 pone-0035380-g008:**
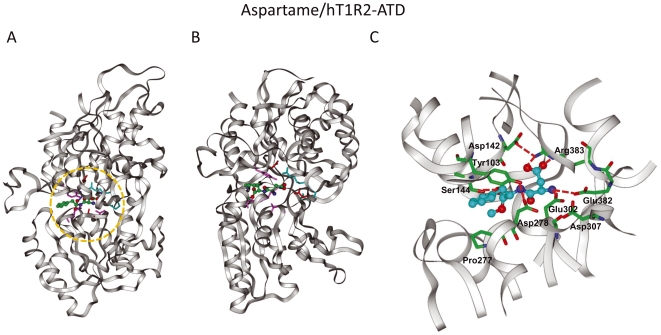
Model of the aspartame-bound hT1R2 ATD. (A) Complex model of aspartame in the closed form of hT1R2 ATD. (B) The model oriented 90° from (A). (C) Aspartame-binding pocket in detail (orange circle in (A)).

To clarify the roles of the 10 residues in small molecular sweetener recognition, we mapped them on the model of the open form of the hT1R2 ATD (Fig. 6). They were divided into four classes based on the results of a single point mutant analysis of hT1R2–hT1R3 corresponding to three chemically different types of ligands: amino acid derivatives (aspartame and d-Trp), sulfamates (saccharin Na and AceK), and a sugar analog (sucralose) (Table 1). Our data strongly suggest that the binding sites in hT1R2 ATD are quite different from each other, although all of them are recognized in the cleft of hT1R2 ATD. As shown in Figs. 7 and 8, aspartame, d-Trp, and sucralose share LB1 residues (Y103 and D142) and LB2 residues (D278, E302, and D307) for binding, but each compound also needs specific residues for individual interaction with the receptor (S144 for aspartame (Fig. 2A) and P277 for sucralose (Fig. 4B)). By contrast, these residues are not involved in binding saccharin Na and AceK, but the residues (D142, E382 and R383) located in another site of LB1 are indispensable for their binding (Figs. 6A).

**Figure 9:**
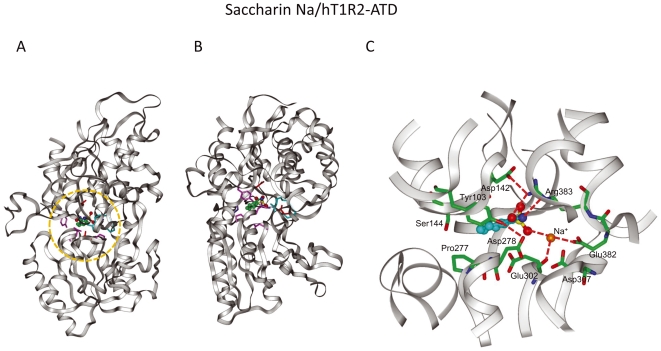
Complex model of the saccharin Na–bound hT1R2 ATD. (A) Complex model of saccharin Na in the closed form of hT1R2 ATD. (B) The model oriented 90° from (A). (C) Saccharin Na–binding pocket in detail (orange circle in (A)). The sodium cation is illustrated by an orange ball.

The low-molecular-weight sweeteners bind in the cleft composed of LB1 and LB2 with a different binding mode at each characteristic residue. To examine further characteristic interactions between ligands and the 10 residues, we built ligand–hT1R2 ATD (closed form) complex models for sucralose, aspartame and saccharin Na (Figs. 7, 8, 9, Methods S1, S2, S3).

### (i) Roles of Y103 at the entry of LB1 and D278 at the entry of LB2

The complex models of sucralose–hT1R2 and aspartame–hT1R2 suggested different roles of Y103 in receptor activation. The C2-H and C4-Cl of the hexose portion of sucralose bind to the aromatic ring of Y103 (Fig. 7C), and the hydroxyl groups in the hexose moiety of sucralose form hydrogen bonds with D278 (Fig. 7C). The binding of the hexose portion to Y103 in LB1 and D278 in LB2 may thus facilitate the formation of the closed form of hT1R2 ATD. The importance of these residues for binding of sucralose is consistent with the results reported by Zhang et al. [18].

Conversely, the phenol group of Y103 forms a hydrogen bond with D278 in the aspartame–hT1R2 model (Fig. 8C), stabilizing the closed form of hT1R2. The hydrogen bond appears to be important for the d-Trp-binding. However, the role of the phenol group in the aspartame-binding would be more significant, since the phenol group would interact with the carboxylate of aspartame. The phenol group of Y103 is thus important for the binding of aspartame, while the aromatic group is necessary for the binding of sucralose, as in the cases of the Y103A and Y103F mutants (Fig. 5A). On the other hand, Zhang et al. [18] suggested a contribution of a hydrogen bond between D278 and K65 to the stabilization of the closed form in the binding of sweet taste enhancers. However, a transiently expressed K65A mutant receptor did not show a significant difference from the native receptor in the binding of aspartame and sucralose (Table S1), being consistent with the results reported by Zhang et al. [18] and Liu et al. [23], in which K65 is not important for the binding of aspartame and sugar derivatives.

### (ii) Roles of E302 at the center of LB2

The negatively charged E302 residue forms a salt bond with the positively charged amine group of aspartame (Fig. 8C), whereas a hydroxyl group of the pentose moiety of sucralose forms a hydrogen bond with E302 (Fig. 7C). E302 in the LB2 should thus be a crucial residue for the ligands, with hydrogen bond donors contributing to the formation of the closed form in receptor activation. In contrast, the E302 residue makes no electrostatic interaction with saccharin Na (Fig. 9C), so the contribution of this residue to receptor activation should be little, if any (Fig. 3B).

### (iii) Roles of D142, E382, and R383 at the center of LB1

Because R383 forms a hydrogen bond network with D142 and E382 in the hT1R2 model, R383 plays a crucial role in the recognition of negatively charged groups of ligands (Fig. 9C). D142 or E382 may not directly interact with the negatively charged ligands but would play an important role in localizing the flexible R383 residue at a proper position for interacting with the ligands (Fig. 9C). For aspartame recognition, binding of both the carboxylate moiety to R383 in LB1 and the amino group to E302 in LB2 may facilitate the formation of the closed form of the ATD (Fig. 8C). The negatively charged group of saccharin and the cationic sodium ion attached to saccharin would play similar roles in the formation of the closed form (Fig. 9C). Liu et al. [23] showed that S40 and V66 contribute to the species specificity in the binding of aspartame. The S40 residue is located at the hydrogen bond distance to D142 and the V66 residue is close to R383 in the aspartame-bound model. The mutation of these residues would electronically and sterically affect the interaction of D142 and R383 which are important for the recognition of the carboxylate of aspartame. This is somewhat similar to the roles of S40 and V66 in the species specific recognition of aspartame.

The neutral ligand sucralose may directly interact with D142 through a hydrogen bond with the vicinal hydroxyl groups of the furanose moiety (Fig. 7C). This hydrogen bond probably leads to the formation of a hydrogen bond between R383 and E302 to facilitate receptor activation.

### (iv) Role of P277 at the entry of LB2

Aspartame and saccharin do not bind P277 (Figs. 7C and 8C). However, aspartame is located near the residue because the Gln mutant for P277 interrupts receptor activation by aspartame. In contrast, the mutation of smaller residues such as Gly and Ser does not affect activation (Fig. 7C). The smaller ligand, saccharin Na, may be located far from P277 and thus may not be influenced by the mutation (Fig. 9C). Still, P277 should be an important binding site for d-Trp, as observed in the P277A and P277Q mutants (Table 1). These results suggest that saccharin Na is located far from P277 whereas d-Trp is located close to P277. The distance between aspartame and P277 would be intermediate between those of saccharin Na and d-Trp.

The chloride at C1' of the furanose moiety of sucralose showed favorable van der Waals contact with P277 (Fig. 7C), and the P277Q mutant caused unfavorable steric interactions with the chloride; however, the favorable hydrophobic interactions are lost in the P277G and P277S mutants (Table 1).

### Characteristic features in receptor activation mechanisms of the human sweet taste receptor

As described above, the interaction at the core of LB1 and LB2 appears to be essential for reception of all the sweeteners, and the interaction at the entry of LB1 and LB2 would reinforce the formation of the closed structure of the receptor for activation. These results strongly suggest that the activation mechanism of the human sweet taste receptor is similar to that of mGluR1.

X-ray crystal structural analysis, molecular modeling, and many mutagenesis studies have revealed the existence of critical residues for ligand binding in other class C GPCRs, such as mGluRs [15], [24], [25], the GABA receptor [26], [27], the calcium sensing receptor [28], [29], and the human umami taste receptor (hT1R1–hT1R3) [30]. In comparison with previous data [31], our model of hT1R2–hT1R3 based on a mutagenesis analysis suggests that hT1R2–hT1R3 uses five acidic residues (D142, D278, E302, D307, or E382) for the recognition of its agonists; the other receptors use one or two acidic residues. These results suggest that hT1R2 ATD forms different sites of binding with specific sets of these residues to receive chemically diverse low-molecular-weight sweeteners, although their affinities for hT1R2 ATD are quite low.

It should be noted that we could not determine the binding mode of sugars such as sucrose. Sugars generally elicit the strong sweet taste, and they are the most common natural ligands for the receptor. Although it would be important to elucidate the key residues for the recognition of sugars, the cellular response to sucrose was quietly faint compared with the other sweeteners used in this study, and EC_50_ values of the mutated receptors to sucrose could not be accurately calculated. Further studies should be required to improve the sensitivity of the functional assay system for the human sweet taste receptor.

In this study, we defined how hT1R2–hT1R3 acquires the ability to recognize chemically diverse sweeteners. These results will not only provide insights into molecular recognition patterns of GPCRs but may also help develop novel sweeteners.

## Supporting Information

Table S1 **Summary of point mutations determined by a calcium imaging assay using HEK293T cells transiently expressing the T1R2 mutant and T1R3.**
(DOC)

Figure S1 **Sequence alignment of the ATDs of hT1R2 and rat mGluR1.** The mutated residues in hT1R2 used for initial screening are shown in blue and magenta. Stable cell lines were also constructed for the residues shown in magenta. Critical ligand-binding residues in the rat mGluR1 ATD that interact with the carboxylate side chain and the α-amino acid moiety are shown in red and green, respectively.
(TIF)

Methods S1 **Modeling for sucralose-T1R2ATD complex (** **Fig. 7A** **–** [Fig pone-0035380-g008] **9C** **).**
(DOC)

Methods S2 **Modeling for aspartame-T1R2ATD complex (** **Fig. 8A–C** **).**
(DOC)

Methods S3 **Modeling for saccharin-T1R2ATD complex (** **Fig. 9A–C** **).**
(DOC)
