# Supplementary material for: Characterization of the Modes of Binding between Human Sweet Taste Receptor and Low-Molecular-Weight Sweet Compounds
Source: PLoS One. 2012 Apr 20;7(4):e35380. doi: 10.1371/journal.pone.0035380 (PMC3335050; doi:10.1371/journal.pone.0035380)
Supplement: Table S1 — Summary of point mutations determined by a calcium imaging assay using HEK293T cells transiently expressing the T1R2 mutant and T1R3. (DOC) [file pone.0035380.s001.doc]

Supplemental table 1. Summary of point mutations determined by a calcium imaging assay using HEK293T cells transiently expressing the T1R2 mutant and T1R3.

The responses were examined by a calcium imaging assay using HEK293T cells transiently expressing the T1R2 mutant and T1R3. Each column indicates the responsiveness of the mutant to the sweetener. Symbols used are as follows: ++, intense response as same as that of WT; +, moderate response; ±, weak or faint response; and -, no response. WT, wild type.

|  |  | aspartame | D-tryptophan | saccharin Na | acesulfame K | sucralose | cyclamate |
| --- | --- | --- | --- | --- | --- | --- | --- |
|  | WT | ++ | ++ | ++ | ++ | ++ | ++ |
| S40 | S40A | - | ± | ± | ± | ± | ± |
| K65 | K65A | ++ | ++ | ++ | ++ | ++ | ++ |
| Y103 | Y103A | - | + | ++ | ++ | + | ++ |
|  | Y103F | + | + | + | + | ++ | ++ |
| D142 | D142A | - | ± | ± | ± | + | + |
|  | D142R | - | - | - | - | - | - |
| S144 | S144A | - | + | ++ | ++ | ++ | ++ |
| S165 | S165A | ++ | + | ++ | ++ | ++ | ++ |
| Y215 | Y215A | - | - | - | - | - | - |
|  | Y215F | + | ± | + | + | + | + |
| P277 | P277A | + | + | + | + | + | + |
|  | P277G | ++ | + | ++ | ++ | + | ++ |
|  | P277S | ++ | ++ | + | + | ++ | + |
|  | P277Q | ± | + | + | + | + | + |
| D278 | D278A | + | + | ++ | ++ | ± | ++ |
| Y282 | Y282A | + | + | + | + | + | + |
| E302 | E302A | - | ± | + | + | ++ | ++ |
| S303 | S303A | ++ | ++ | ++ | ++ | ++ | ++ |
| D307 | D307A | + | + | + | + | ++ | + |
| E382 | E382A | ± | + | ± | ± | + | + |
| R383 | R383A | - | ± | - | - | ± | - |
|  | R383D | - | - | - | - | ± | - |
|  | R383Q | - | ± | - | - | ± | ± |
|  | R383L | - | - | - | - | - | - |
|  | R383H | + | ++ | + | + | ++ | + |
