# Supplementary figures and images for: Characterization of the Modes of Binding between Human Sweet Taste Receptor and Low-Molecular-Weight Sweet Compounds
Source: PLoS One. 2012 Apr 20;7(4):e35380. doi: 10.1371/journal.pone.0035380 (PMC3335050; doi:10.1371/journal.pone.0035380)

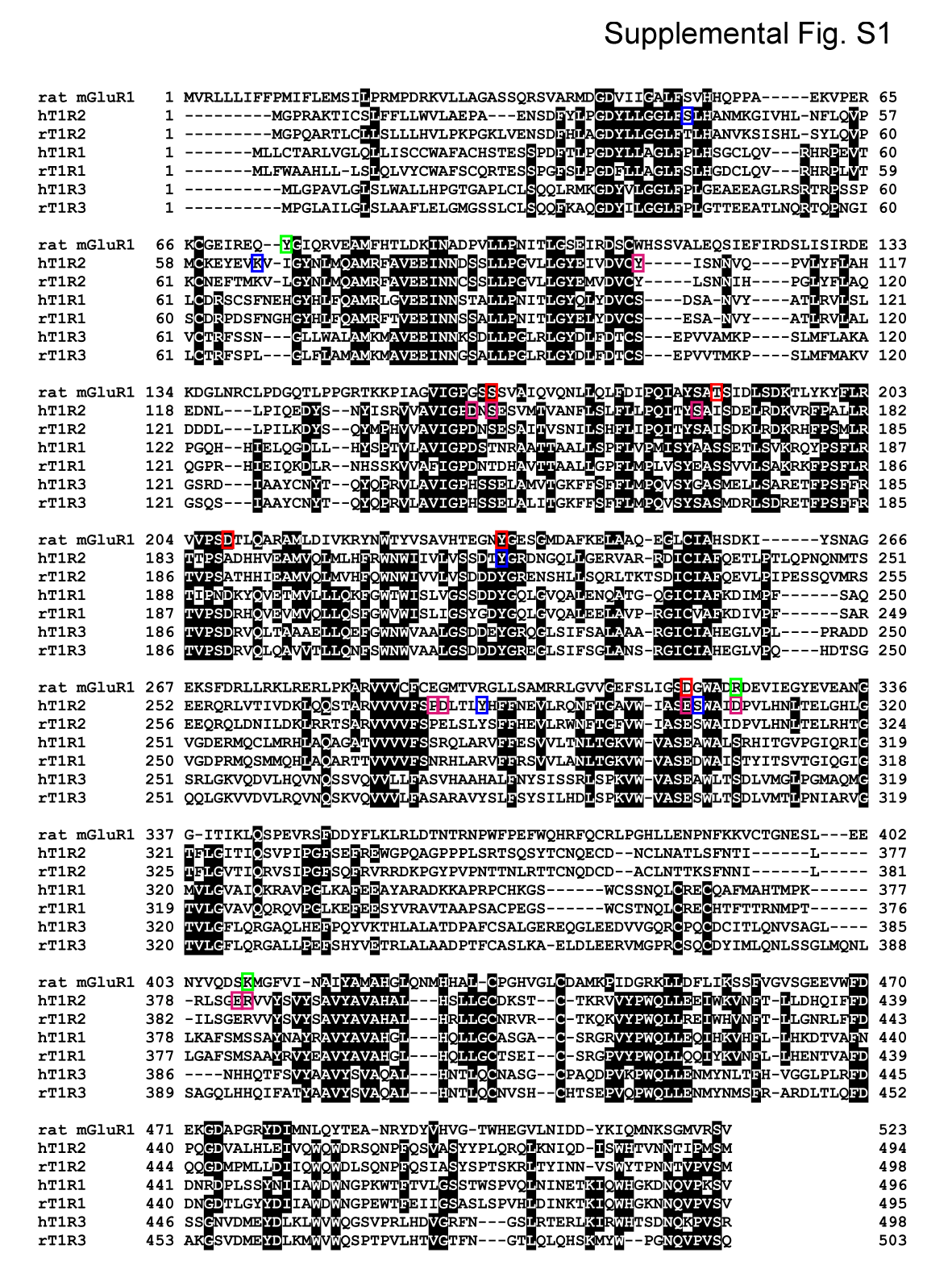

Supplement: Figure S1 — Sequence alignment of the ATDs of hT1R2 and rat mGluR1. The mutated residues in hT1R2 used for initial screening are shown in blue and magenta. Stable cell lines were also constructed for the residues shown in magenta. Critical ligand-binding residues in the rat mGluR1 ATD that interact with the carboxylate side chain and the α-amino acid moiety are shown in red and green, respectively. (TIF) [file pone.0035380.s002.tif]
