# Supplementary material for: Characterization of the Modes of Binding between Human Sweet Taste Receptor and Low-Molecular-Weight Sweet Compounds
Source: PLoS One. 2012 Apr 20;7(4):e35380. doi: 10.1371/journal.pone.0035380 (PMC3335050; doi:10.1371/journal.pone.0035380)
Supplement: Methods S1 — Modeling for sucralose-T1R2ATD complex ( Fig. 7A – 9C ). (DOC) [file pone.0035380.s003.doc]

**Methods S1** Modeling for sucralose-T1R2ATD complex (Fig. 7A-C)

Sucralose can assume two possible binding forms. One involves the binding of the furanose moiety at the center of the LB1 and LB2 cleft, the other the pyranose moiety in the center. After modeling two complexes, the former binding form appeared to be responsible for the results of the mutation experiments. Thus, the furanose moiety was bound in the center of the cleft at a plausible hydrogen-bond distance, between the vicinal diol moiety and E302. Then a hydrogen bond was formed between the other vicinal diol in the pyranose moiety and the two carboxylates, D278 and D307. The final structure was optimized by molecular dynamics calculations, after which the complex structure showed another hydrogen bond between the primary hydroxyl group at the C6 position of the pyranose and S142. In addition, the pyranose moiety was laid on the aromatic ring of Y103, and the chloromethyl group was located nearby at P277 with a hydrophobic interaction.
