# Supplementary material for: Characterization of the Modes of Binding between Human Sweet Taste Receptor and Low-Molecular-Weight Sweet Compounds
Source: PLoS One. 2012 Apr 20;7(4):e35380. doi: 10.1371/journal.pone.0035380 (PMC3335050; doi:10.1371/journal.pone.0035380)
Supplement: Methods S2 — Modeling for aspartame-T1R2ATD complex ( Fig. 8A–C ). (DOC) [file pone.0035380.s004.doc]

**Methods S2** Modeling for aspartame-T1R2ATD complex (Fig. 8A-C)

We have assumed that the positively charged amino group binds at E302 and found the positively charged R383 residue can interact with the carboxylate of aspartame. These interactions led us to find another important interaction between the ester carbonyl moiety and S144. The final structure was optimized by molecular dynamics calculations, and thus the phenylalanine moiety was near Y103 and P277. However, the aromatic group did not show a specific interaction with Y103 and P277. In contrast, the phenolic hydroxyl group of Y103 formed a hydrogen bond with the carboxylate of D278. The carboxylate of aspartame formed a hydrogen bond with R383 and R383 formed hydrogen bonds with D142 and E382, located proximal to the cationic amine moiety of aspartame within hydrogen-bonding distance.
