# Supplementary material for: Characterization of the Modes of Binding between Human Sweet Taste Receptor and Low-Molecular-Weight Sweet Compounds
Source: PLoS One. 2012 Apr 20;7(4):e35380. doi: 10.1371/journal.pone.0035380 (PMC3335050; doi:10.1371/journal.pone.0035380)
Supplement: Methods S3 — Modeling for saccharin-T1R2ATD complex ( Fig. 9A–C ). (DOC) [file pone.0035380.s005.doc]

**Methods S3** Modeling for saccharin-T1R2ATD complex (Fig. 9A-C)

The negatively charged sulfamate moiety was located at the ionic bond distance from R383. After location of the sodium ion near the sulfamate moiety, the final structure was optimized by molecular dynamics calculations, the amide oxygen and nitrogen of the sulfamate moiety formed hydrogen bonds with R383, and the sodium ion formed an ionic bridge between a sulfate oxygen and the carboxylate of E382. The phenyl group of saccharin was located far from P277, and thus the P277Q mutant appeared not able to affect saccharin binding (Table 1B).
